# Agricultural exposures and DNA damage in PBMC of female farmers measured using the alkaline comet assay

**DOI:** 10.1007/s00420-024-02049-z

**Published:** 2024-03-02

**Authors:** P. Evenden, Q. Vandoolaeghe, Y. Lecluse, A. C. Gac, R. Delépée, L. B. Weiswald, E. Boutet-Robinet, M. Boulanger, S. Bonassi, P. Lebailly, M. Meryet-Figuière

**Affiliations:** 1 Inserm U1086 ANTICIPE (Interdisciplinary Research Unit for Cancer Prevention and Treatment), Normandie Univ, Université de Caen Normandie, Caen, France; 2 Comprehensive Cancer Center François Baclesse, UNICANCER, Caen, France; 3 Toxalim (Research Centre in Food Toxicology), INRAE, ENVT, INP-Purpan, UPS, Université de Toulouse, Toulouse, France; 4 Department of Human Sciences and Quality of Life Promotion, San Raffaele University, Rome, Italy; 5 Unit of Clinical and Molecular Epidemiology, IRCCS San Raffaele Roma, Rome, Italy

**Keywords:** Occupational health, Comet assay, DNA damage, Agriculture, Female workers

## Abstract

**Objective:**

Several studies investigated the link between agricultural occupational exposures and DNA damage, in an attempt to bring elements of biological plausibility to the increased cancer risk associated with them. However, only a few of these studies focused on females.

**Methods:**

The comet assay was performed on PBMC (Peripheral Blood Mononuclear Cells) samples from 245 females working in open field farming and cattle raising, located in the Normandy area of France. Individual questionnaires on tasks performed were administered at the time of sampling to directly assess exposures. Environmental exposures were issued from a questionnaire assessing the farm productions. Linear regression analyses were done using the DNA damage scores.

**Results:**

Regarding direct exposures, several tasks associated with exposure to potentially harmful chemicals were not associated with DNA damage, but a longer duration of use of herbicide on meadows (*p* = 0.05) or of cleaning and upkeep of agricultural equipment (*p* = 0.06) revealed higher DNA damage levels, although the number of exposed women was low. Several indirect and/or environmental exposures were associated with DNA damage in multivariate analyses: a larger surface of meadows (*p* = 0.006) or the presence of poultry (*p* = 0.03) was associated with less DNA damage, while the presence of swine (*p* = 0.01) was associated with higher DNA damage. Smokers and former smokers had less DNA damage than non-smokers (*p* = 0.0008 and *p* = 0.03).

**Conclusions:**

We report modified levels of DNA damage for those environmentally exposed to meadows, poultry and pig farming, underlining the need for a better knowledge of the potential health risks experienced by females in this setting.

**Supplementary Information:**

The online version contains supplementary material available at 10.1007/s00420-024-02049-z.

## Introduction

Farming exposures have been associated with an increased risk of many diseases, of which cancer is most often reported (Lemarchand et al. 2017; Lerro et al. 2019). Farming covers a wide range of exposures and activities. Numerous studies identified associations between increased cancer risk and direct exposure to pesticides through application (Schinasi and Leon 2014; Lewis-Mikhael et al. 2016), or indirectly through re-entry or harvesting (Piel et al. 2017; Tual et al. 2019).

However, the underlying biological mechanisms linking exposures with cancer risk remain insufficiently characterized. A better understanding of these mechanisms could help to define adapted preventive action and identify harmful exposures before cancer diagnosis. Also, it would provide clues about the biological plausibility of the link between exposures and cancer risk, advancing our understanding of cancer etiology (Vineis and Perera 2007).

Biomarkers of genotoxicity or related to genome stability are linked with cancer development, and have been associated with pesticide exposure in different settings (Bolognesi 2003). Perturbations of DNA methylation (Alexander et al. 2017), an increase of micronuclei (Bolognesi et al. 2011), and of DNA damage measured by the comet assay (Lebailly et al. 2015; Nascimento et al. 2022) are present in farmers exposed to pesticides.

Most of these studies are however focused on men or else women represent only a small fraction of the studied population mostly because women are seldom directly exposed to pesticides through spraying in fields (Dahiri et al. 2021), although they are environmentally exposed while living on farms (Deziel et al. 2015), or through care of farm animals. However, the few studies aiming to evaluate genotoxicity in women indirectly exposed to pesticides report increased micronuclei rates for female farmers overall (Castañeda-Yslas et al. 2016) and increased DNA damage by comet assay for females working in cotton fields (Perumalla Venkata et al. 2017; Ali et al. 2018), plucking tea leaves (Dhananjayan et al. 2019), or working in greenhouses (Cayir et al. 2019). Therefore, although their exposure is to be different from men, as is their biology, the consequences of farming exposures in women ought to be monitored to better assess and understand the risks they are exposed to.

The comet assay in alkaline conditions is able to detect single strand and double-strand DNA breaks, as well as abasic and alkali-labile sites. It has been long used to successfully monitor DNA damage in biomonitoring studies, not only in agricultural settings (Silva Pinto et al. 2020; Milić et al. 2021; Nascimento et al. 2022).

Our aim with this study was to assess DNA damage with comet assay and study their association with occupational and environmental exposures in a cohort of women involved in farming. This study is to our knowledge the first of its kind by reporting DNA damage in over 200 women with regard to agricultural exposures.

## Materials and methods

### Farm and subjects selection

**Fig. 1:**
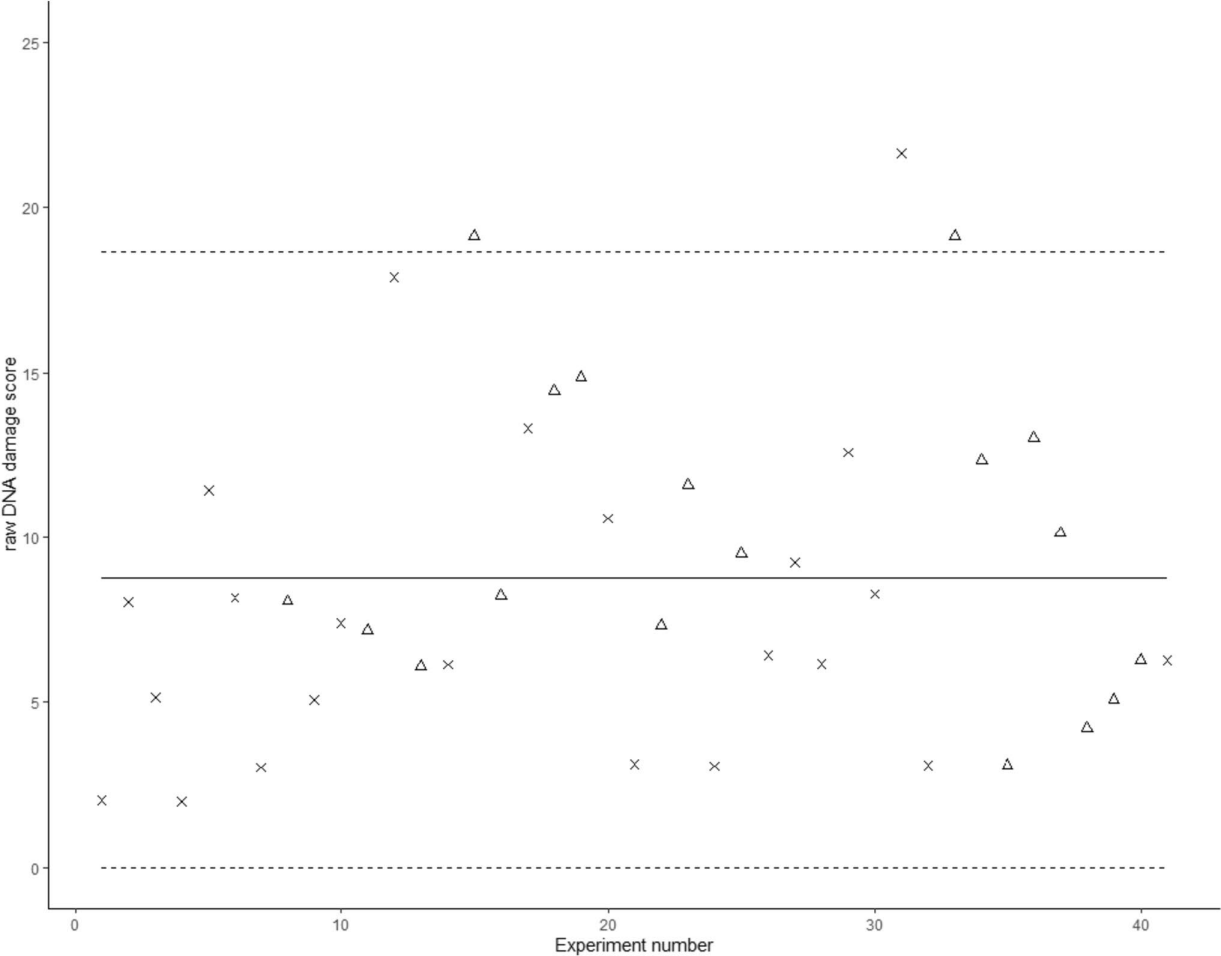
Variability of internal standard for the 41 experiments. The solid line represents the mean (8.77), the two dotted lines represent the range of mean ± 2SD (±9.90). Triangles (experimenter 1) or crosses (experimenter 2) represent the experiments performed by 2 different people

We visited 410 farms in Calvados (France) over the period 1997–2000, where all willing persons (319 females and 443 males gave informed consent) were included in a cohort by completing a questionnaire and donating blood samples. This cohort was described by Roulland et al. (2004). Ethical approval was obtained from the ethical committee (Comité Consultatif Pour les Personnes Se Prêtant à la Recherche Biomédicale #99-07, Caen, France). The questionnaire contained information regarding lifetime occupational exposure (use of pesticides, tasks undertaken on the farm) as well as socio-demographic information and individual habits. A second survey detailing farm characteristics and agricultural activities was also filled out for each farm (Utilized Agricultural Land, crops and livestock found on the premises, equipment). Information on all female participants is shown in a flow chart (Fig. 1).

### PBMC samples

From heparinized blood samples, peripheral blood mononuclear cells (PBMCs) were separated by Ficoll Hypaque gradient centrifugation (GE Healthcare) following manufacturer’s instructions. The PBMCs obtained were then suspended in a solution of RPMI1640 (Gibco) with 20% FBS (Fetal Bovine Serum, heat-inactivated in all procedures) (Gibco) and 10% DMSO (Dimethyl sulfoxide) (Euromedex) to ensure long-term viability of cells in liquid nitrogen.

### Standardization method

For each experiment, a historical control was integrated to assess the potential intra- and inter-experimental variability. This control sample was constituted of PBMCs from blood drawn from a healthy volunteer unexposed to agriculture. One aliquot of this control was used per experiment to avoid freeze–thaw cycles.

### Comet assay

Cells were quickly thawed at 37 °C in a water bath. The aliquot was re-suspended in 6 mL of RPMI1640 with 10% FBS and centrifuged at 200 RCF for 10 min. The pellet was re-suspended in 1 mL of RPMI1640 with 10% FBS. The viability of the cells was tested with Trypan blue. Samples with <90% viability were excluded (3.5% of the 254 samples available, Fig. 1). Cells were brought to a concentration of 120,000 cells/mL in RPMI1640 with 10% FBS and centrifuged at 200 RCF for 10 min.

We then followed the protocol described by Perdry et al. (2018), although with a reduced throughput: in each experiment, we used a Gelbond® (GE Healthcare) sheet holding 10 samples in duplicate and a duplicate of the historical control.

The methodology we used is in accordance with the Minimum Information for Reporting on the Comet Assay (MIRCA) recommendations (Møller et al. 2020).

### Comet scoring

We acquired a virtual image of each slide using a fluorescence scanner (VS120, Virtual Slide Microscope, Olympus Life Science).

For each gel deposit, 100 randomly selected cells were visually analyzed (200 cells per sample). They were graded into four categories according to a visual scoring system: undamaged, lightly damaged, moderately damaged and highly damaged cells. We assigned a rank number ranging from 0 to 3 to each category.

Some studies state that highly damaged cells (HDCs) are not all apoptotic cells (Lorenzo et al. 2013), while others indicate that HDCs are rather dying (Collins et al. 2008). Therefore, at least a fraction of HDCs would be apoptotic cells, which are not segregated by the comet assay. We thus ran our analyses first without HDCs to exclude DNA damage resulting from apoptosis in dying cells.

The DNA damage score per deposit was expressed as Eq. 1. Both deposit scores were then added together and normalized from the standardization control of their experiment.

1 $${S}_{{\text{deposit}}}=\frac{{n}_{0}\times 0+{n}_{1}\times 1+{n}_{2}\times 2}{{n}_{0}+{n}_{1}+{n}_{2}}\times 100$$

In the sensitivity analysis, the comet damage score included HDCs as per Eq. 2.

2 $${S}_{{\text{deposit}},\mathrm{ HDC}}={n}_{0}\times 0+{n}_{1}\times 1+{n}_{2}\times 2+{n}_{3}\times 3$$

All scoring was blinded and carried out by a trained researcher.

### Standardization

By analyzing the data from the 41 experiments ran, the mean damage score was of 8.77 with a SD of 4.95 (range 2.0–21.64) (Supplementary Table S1). The variability of damage scores does not show a trend over time (Fig. 1), and with the exception of 3 experiments falls within 2 SD, which is acceptable for comet assay (De Boeck et al. 2000). The removal of the 3 experiments does not change our results (data not shown). Two people performed the experiments, and no deviation in scores appear between these (Fig. 1). We normalized the comet scores of the samples by dividing the comet score by the score of its nested control.

### Statistical analysis

The study population was described by mean and standard deviation when the data was normally distributed. For other continuous variables, median and IQR were expressed. Categorical variables were described by proportion. For categorical variables, *χ*^2^ or Fisher exact tests were used when appropriate. For continuous variables, Student t test or Wilcoxon non-parametric test was used when appropriate. Explanatory variables were selected based on the number of observations (excluded if *n* < 5). Complete case analysis was performed, all selected variables had <5% missing data (bar one at 6%). Comet DNA damage score was log-transformed, which allowed a normal distribution. To test associations between explanatory variables and the log-transformed comet score, univariable linear regression analyses were performed. For occupational and environmental farmland exposures, individuals unexposed to the considered exposure were taken as a reference. For all tested associations between a variable and DNA damage, we considered that a *p* value below 0.2 was worth being reported. Such associations are termed as “tendencies” and referred to accordingly in the manuscript. Associations with a *p* value below 0.05 are reported as “associations” and are referred to accordingly in the manuscript. Our approach to analyze our findings beyond the classical view of statistical significance is in accordance with recommendations (Amrhein et al. 2019). For multivariable analysis, all variables associated with DNA damage with a *p* value below 0.20 were included in a first multivariable model. A backwards selection was performed to obtain a final multivariable model. Sensitivity analyses were run by introducing the HDCs. The statistical analysis was generated using SAS software, Version 9.4 of the SAS System for Windows.

## Results

### Study population and sample selection

**Fig. 2:**
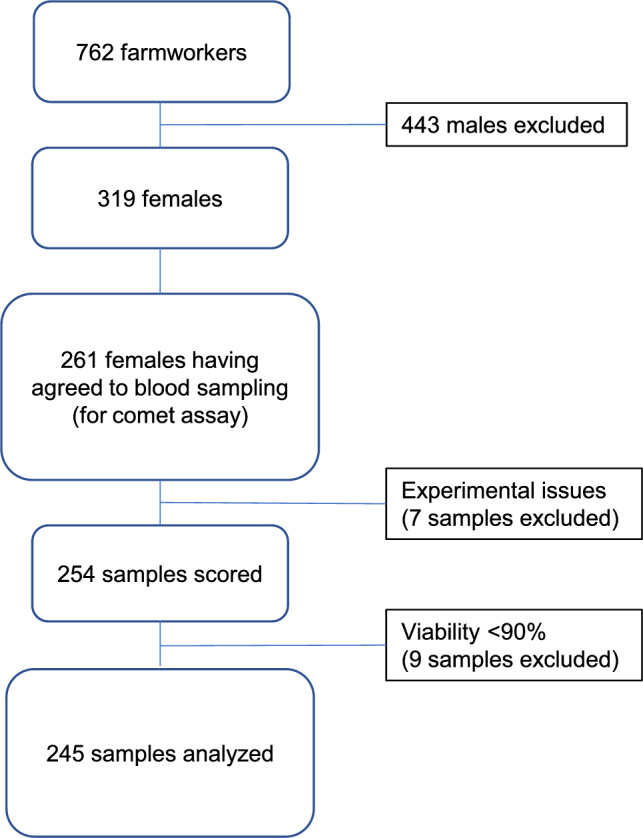
Flow chart of sample selection

Our sample selection is described with the flow chart in Fig. 2. Of the 319 females included, 261 gave blood samples, of which 254 could be used for the comet assay. Nine samples were excluded due to insufficient cell viability. The results presented in this study rely on 245 female samples.

### Socio-demographic characteristics associated to DNA damage

Mean age of the 245 females analyzed was 46 years old at enrollment (ranging from 19 to 73 years), 58% of them were of normal BMI (18.5–25 kg/m^2^). The majority of the females were never smokers (80%) and 45% consumed alcohol occasionally (Table 1).

**Table 1:** Univariable analyses between socio-demographic characteristics and DNA damage

Variable	Females analyzed through the comet assay (*n* = 245)	Median comet score (IQR)	Β coefficient ± SD	*p* value
Age	Mean ± SD			
	45.74 ± 10.63	13.19 (13.94)	0.009 ± 0.01	0.80
BMI (classes)	*n*, (%)			
18.5	5 (2.05)	15.08 (12.60)	−0.32 ± 0.50	0.52
[18.5–25]	142 (58.20)	12.99 (16.07)	Ref	
≥25	97 (39.75)	13.72 (12.69)	0.21 ± 0.14	0.15
Missing	1	1		
Smoking status	*n*, (%)			
Current smoker	23 (9.39)	10.36 (6.48)	−0.63 ± 0.24	0.01^*^
Former smoker	27 (11.02)	12.40 (13.04)	−0.38 ± 0.22	0.09^†^
Never smoker	195 (79.59)	14.07 (15.98)	Ref	
Missing	0	0		
Current smokers: cigarettes/day, *n* = 20, median (IQR)	9 (16.5)	10.39 (6.05)	−0.037 ± 0.0207	0.09^†^
Current and ex−smokers: Cigarettes/day, *n* = 45	6 (12)	10.87 (11.08)	−0.020 ± 0.018	0.27
Alcohol consumption	*n*, (%)			
None	51 (20.82)	15.03 (17.22)	Ref	
Occasional	110 (44.89)	11.58 (12.20)	−0.08 ± 0.19	0.68
Daily	82 (33.74)	13.70 (13.91)	−0.12 ± 0.20	0.53
Missing	2	2		

^*^p < 0.05; ^†^*p* < 0.2

There was no variation in DNA damage levels with age, BMI, or alcohol consumption. Smoking at the time of sampling was associated with a decrease in DNA damage compared with no smoking history (*p* = 0.01). Former smoking revealed a tendency of decreased DNA damage compared with no smoking history (*p* = 0.09). We observed a trend of decreased DNA damage in association with smoking and former smoking (*p* = 0.01). A tendency of decreased DNA damage was found with an increased quantity of cigarettes smoked per day (*p* = 0.09) (Table 1), only when current smokers are compared to ex- and never-smokers together.

### Occupational exposure associated to DNA damage

Participants undertaking tasks associated with crops were lowly represented, where the most implication was for harvest work with 34 women (14%). The use of herbicides on meadows was not associated with differences in DNA damage levels (*p* = 0.68). However, a longer duration of use of herbicides on meadows for people undertaking this task show a tendency of higher DNA damage (*n* = 5, *p* = 0.05). Cleaning and upkeep of agricultural equipment was not associated with modified DNA damage levels (*p* = 0.65), but a longer duration of practicing this task showed a tendency to have higher DNA damage levels (*n* = 18, *p* = 0.06). Regarding tasks related to livestock, people undertaking milking tended to have higher DNA damage levels (*p* = 0.16) (Table 2).

**Table 2:** Univariable linear regression for occupational exposure and DNA damage

Exposure variable		Median comet score (IQR)	*β* coefficient ± SD	*p* value
*Crops*				
Plowing work, *n* (%)	9 (3.67)	19.15 (19.96)	−0.01 ± 0.38	0.99
Sewing work, *n* (%)	7 (2.86)	9.33 (20.91)	0.20 ± 0.42	0.64
Harvest work, *n* (%)	34 (13.88)	13.39 (11.77)	−0.06 ± 0.21	0.77
Seed treatment, *n* (%)	5 (2.04)	6.12 (8.11)	0.06 ± 0.50	0.90
Storage silo treatment, *n* (%)	6 (2.45)	10.90 (13.14)	−0.04 ± 0.46	0.93
Use of herbicides on meadows, *n* (%)	5 (2.04)	8.72 (5.59)	0.21 ± 0.50	0.68
Duration of use, median (IQR), *n* = 5	4 (7)		0.07 ± 0.02	0.05^*^
*Livestock*				
Care of livestock, *n* (%)	159 (64.90)	13.19 (13.31)	−0.12 ± 0.16	0.43
Use of hormones, *n* (%)	19 (7.76)	15.08 (13.47)	−0.10 ± 0.27	0.69
Use of antiparasites and insecticides on livestock, *n* (%)	138 (56.33)	13.07 (13.11)	−0.16 ± 0.14	0.24
Duration of use, median (IQR), *n* = 126	22 (15)	13.03 (12.94)	−0.005 ± 0.01	0.56
Milking, *n* (%)	109 (44.49)	14.58 (17.11)	0.20 ± 0.15	0.16^†^
Disinfection of milking equipment, *n* (%)	117 (47.76)	14.07 (14.54)	0.09 ± 0.14	0.50
Duration of use, median (IQR), *n* = 112	21.5 (11)	14.33 (14.95)	0.002 ± 0.01	0.87
*Other*				
Driving of other machinery, *n* (%)	7 (2.86)	15.08 (17.45)	−0.49 ± 0.42	0.25
Use of rodent poison (raticide, etc.), *n* (%)	92 (37.55)	13.12 (14.55)	−0.07 ± 0.15	0.65
Use of herbicides on courtyards, *n* (%)	113 (46.12)	12.87 (13.15)	0.004 ± 0.14	0.98
Duration of use, median (IQR), *n* = 103	19 (14)	12.92 (13.69)	0.01 ± 0.01	0.44
Use of herbicides on embankments, *n* (%)	32 (13.06)	12.93 (13.32)	0.04 ± 0.21	0.83
Duration of use, median (IQR), *n* = 32	15.5 (11.5)		−0.01 ± 0.02	0.58
Disinfection of premises and facilities, *n* (%)	94 (38.37)	13.76 (15.03)	0.03 ± 0.14	0.83
Duration of use, median (IQR), *n* = 80	21 (14)	13.68 (15.03)	0.007 ± 0.01	0.54
Cleaning and upkeep of agricultural equipment, *n* (%)	18 (7.35)	13.42 (15.16)	0.12 ± 0.27	0.65
Duration of use, median (IQR), *n* = 18	13.5 (13)		0.06 ± 0.03	0.06^†^

^*^p < 0.05; ^†^*p* < 0.2

### Environmental farmland exposure associated to DNA damage

Farm characteristics were assessed by a separate questionnaire which was filled in by the farm owner, describing what is present on the farm regardless of individual activities, therefore assessing the general working and living environment participants are exposed to.

Increased utilized agricultural area (UAA) overall on the farm was associated with lower levels of DNA damage (*p* = 0.01).

Regarding specific crops, no modification of DNA damage levels was found in people working on farms where rape, meadows or wheat crops are present (rape *p* = 0.64, meadows *p* = 0.93 and wheat *p* = 0.56 respectively). However, an increased area of rape and meadows were associated with reduced levels of DNA damage (*p* = 0.04 and *p* = 0.03 respectively), there was also a tendency of reduced DNA damage levels with increased area of wheat crops (*p* = 0.07).

No association was visible with the presence of orchards (*p* = 0.98), but DNA damage levels tended to be higher with increasing orchard size (*p* = 0.19).

The presence of flax and pea crops on the farm led to a tendency to lower DNA damage (*p* = 0.18 and *p* = 0.15). However, size of these crops was not associated with modified DNA damage levels (*p* = 0.34 for flax and *p* = 0.33 for pea).

Concerning livestock, the presence of sheep, cattle, and goats was not associated with modification of DNA damage levels (sheep *p* = 0.62, cattle *p* = 0.82, goats *p* = 0.53). However, increasing number of sheep on the farm was associated with reduced DNA damage levels (*p* = 0.04), and there was also a tendency to reduced DNA damage levels with the number of cattle and goats (*p* = 0.17 and *p* = 0.06, respectively) (Table 3).

**Table 3:** Farm characteristics and univariable association with DNA damage score

Variable		Median comet score (IQR)	*β* coefficient ± SD	*p* value
Utilized agricultural area (ha), median (IQR)	68 (65)	13.19 (13.94)	−0.003 ± 0.001	0.01^*^
Wheat				
Presence, *n* (%)	172 (70.20)	13.43 (14.46)	−0.09 ± 0.15	0.56
Area (ha) (*n* = 172), median (IQR)	15.5 (23.25)		−0.006 ± 0.003	0.07^†^
Barley				
Presence *n* (%)	102 (41.63)	14.07 (13.34)	0.05 ± 0.14	0.71
Area (ha) (*n* = 102), median (IQR)	5.25 (7.50)		−0.002 ± 0.01	0.91
Corn				
Presence *n* (%)	153 (62.45)	13.60 (14.67)	0.01 ± 0.15	0.92
Area (ha) (*n* = 153), median (IQR)	15 (10.5)		−0.01 ± 0.01	0.53
Peas				
Presence *n* (%)	69 (28.16)	12.84 (14.39)	−0.21 ± 0.16	0.18^†^
Area (ha) (*n* = 69), median (IQR)	12 (17)		−0.01 ± 0.01	0.33
Beets				
Presence *n* (%)	50 (20.41)	11.88 (17.27)	−0.05 ± 0.17	0.78
Area (ha) (*n* = 50), median (IQR)	4.25 (6)		−0.005 ± 0.03	0.86
Flax				
Presence *n* (%)	21 (8.57)	13.37 (14.71)	−0.36 ± 0.25	0.15^†^
Area (ha) (*n* = 21), median (IQR)	6 (5)		0.02 ± 0.02	0.34
Rape				
Presence *n* (%)	37 (15.10)	15.05 (12.67)	−0.02 ± 0.20	0.93
Area (ha) (*n* = 37), median (IQR)	5 (7)		−0.06 ± 0.03	0.04^*^
Potato				
Presence *n* (%)	9 (3.67)	8.73 (5.21)	−0.29 ± 0.37	0.44
Area (ha) (*n* = 9), median (IQR)	5 (13)		−0.01 ± 0.06	0.82
Orchard				
Presence *n* (%)	13 (5.31)	13.37 (12.69)	0.01 ± 0.31	0.98
Area (ha) (*n* = 13), median (IQR)	10 (14)		0.02 ± 0.02	0.19^†^
Meadows				
Presence *n* (%)	220 (89.80)	13.06 (13.87)	−0.11 ± 0.23	0.64
Area (ha) (*n* = 220), median (IQR)	25 (27)		−0.007 ± 0.003	0.03^*^
Livestock farming				
Presence *n* (%)	239 (97.55)	13.19 (13.90)	−0.12 ± 0.45	0.78
Dairy				
Presence *n* (%)	149 (60.82)	13.60 (14.45)	0.07 ± 0.15	0.64
Cattle				
Presence *n* (%)	207 (84.49)	13.19 (13.78)	−0.05 ± 0.19	0.82
Number of animals (*n* = 207), median (IQR)	100 (85)		−0.001 ± 0.001	0.17^†^
Sheep				
Presence *n* (%)	57 (23.27)	13.40 (12.85)	−0.08 ± 0.17	0.62
Number of animals (*n* = 57), median (IQR)	5 (7)		−0.006 ± 0.003	0.04^*^
Pig				
Presence *n* (%)	41 (16.73)	14.07 (16.96)	0.38 ± 0.19	0.04^*^
Number of animals (*n* = 41), median (IQR)	2 (2)		−0.00003 ± 0.0005	0.95
Horse				
Presence *n* (%)	49 (20.00)	14.63 (10.54)	−0.05 ± 0.18	0.77
Number of animals (*n* = 49), median (IQR)	3 (9)		0.006 ± 0.01	0.54
Goat				
Presence *n* (%)	17 (6.94)	12.40 (10.93)	0.18 ± 0.28	0.53
Number of animals (*n* = 17), median (IQR)	1 (1)		−0.30 ± 0.15	0.06^†^
Rabbit				
Presence *n* (%)	98 (40.00)	13.53 (12.74)	0.14 ± 0.14	0.33
Number of animals (*n* = 98), median (IQR)	10 (11)		−0.0002 ± 0.0003	0.56
Poultry				
Presence *n* (%)	183 (74.69)	13.04 (14.05)	−0.23 ± 0.16	0.15^†^
Number of animals (*n* = 183), median (IQR)	30 (35)		0.000006 ± 0.00001	0.56

^*^p < 0.05; ^†^*p* < 0.2

Having pigs present on farms was associated with a higher amount of DNA damage (*p* = 0.04), though the number of pigs was not associated with modified DNA damage levels (*p* = 0.95).

There was a tendency to lower DNA damage when poultry were present on the farm (*p* = 0.15), but without link with the number of animals (*p* = 0.56).

### Multivariable analysis of parameters associated with DNA damage

We ran a multivariate analysis with the parameters with which an association or tendency with modified DNA damage levels, either increase of decrease, was detected. Current and former smoking remained associated with lower DNA damage levels (*p* = 0.0008 with *β* = −0.80 and *p* = 0.03 with *β* = −0.48 respectively), as well as the trend associating smoking with decreased DNA damage (*p* = 0.0009).

No direct occupational exposure remained associated with DNA damage in the multivariate analysis.

Regarding exposures to the farming environment, pig farming was associated with higher DNA damage, (*p* = 0.01) though the presence of poultry farming and the bigger the surface of meadows were associated with lower DNA damage (*p* = 0.003 and *p* = 0.04, respectively) (Table 4).

**Table 4:** Multivariable analysis of exposure and DNA damage

Variable	*β* coefficient ± SD	*p* value
Smoking status		
Current smoker	−0.80 ± 0.24	0.0008^**^
Former smoker	−0.48 ± 0.22	0.03^*^
Never smoker	Ref	
Pig farming (Y/N)	0.45 ± 0.18	0.01^*^
Poultry farming (Y/N)	−0.35 ± 0.16	0.03^*^
Meadows (surface)	−0.008 ± 0.003	0.006^*^

Model: *R*^2^ = 0.1022, *p* < 0.0001

^*^p < 0.05; ^**^p < 0.001

### Analysis of parameters associated to DNA damage including HDCs

We replicated our analysis with HDCs included in the comet score.

The relationship between smoking status and reduced DNA damage remained for current smokers, but as a tendency (*p* = 0.10), and was no longer present for former smokers, nor was the trend of smoking overall (supplementary data table S2).

Concerning occupational exposure, all associations/tendencies remained, although the p values were numerically changed, as could be expected (supplementary table S3).

Concerning farm characteristics, the tendency of lower DNA damage with the presence of flax crops on the farm remained (*p* = 0.16), while it was lost with peas crops. The utilized agricultural area was no longer associated with decreased DNA damage. The tendency of decreased DNA damage with increasing wheat surface disappeared. Increasing size of rape crops and meadows continued to be associated with lower DNA damage, though only as a tendency for meadows (*p* = 0.04 and *p* = 0.14 respectively). The tendency of higher DNA damage with the presence of orchards remained (*p* = 0.19), as it was with the presence of pigs (*p* = 0.12), although as a tendency. The presence of poultry however lost its association with DNA damage. Regarding the number of sheep and goats, only the number of sheep continued to be associated with lower DNA damage (*p* = 0.04) (supplementary data table S4).

## Discussion

### Influence of farming professional and environmental exposures on DNA damage

Regarding occupational pesticide exposure, farm workers have an overall increased level of DNA damage compared to unexposed populations (Nascimento et al. 2022) although it depends on the molecules they are exposed to (Lebailly et al. 1998a, 2003).

Studies regarding female agricultural professional exposure to pesticide are scarce likely because women are rarely direct users of pesticides on crops (Dahiri et al. 2021). In our cohort, the low number of women involved in pesticide spraying prevented us to assess its consequences. The repertoire of tasks reported in our study is more detailed than usually reported, and we could report the absence of effect on DNA damage of seed treatment, and the tendency of having increased DNA damage the longer the duration of use of herbicide on meadows, although this was lost in multivariate analysis. However, with *n* = 5, our findings would need to replication in larger cohorts.

There are however several tasks involving the use of potentially harmful chemicals to which more women were exposed, with no association with DNA damage: use of antiparasitic or insecticide on livestock (*n* = 138) or of rodent poison (*n* = 92); use of herbicide on courtyards (*n* = 113) or on embankments (*n* = 32); disinfection of milking equipment (*n* = 117), of premises and facilities (*n* = 94) or the cleaning and upkeep of agricultural equipment (*n* = 18). For this latter task however, there is a tendency to increased DNA damage for a longer duration of practice, which would need further attention in additional studies, although this association is lost in multivariate analysis. Some of these tasks have been associated to an increased risk of cancer in other studies, such as the use of pesticide on livestock with multiple myeloma (Tual et al. 2019), or the disinfection of barns with multiple myeloma or sarcoma (Renier et al. 2022), meaning that either women perform these tasks in a way is less harmful than men, or that the associated cancer risk does not rely on an early increase in DNA damage in PBMC.

Regarding interaction with animals, women involved in milking did present a tendency to increased DNA damage (*p* = 0.16). Although this disappeared in multivariate analysis, it would be worth exploring further this relationship since milking has been associated with an increased risk of sarcoma (Renier et al. 2022), and because this task is involving a large number of women.

Other associations between DNA damage and exposure to animals were present, although in an indirect fashion. The presence of swine on the farm was associated with increased DNA damage, though the presence of poultry was associated with less. To our knowledge, literature shows no previous studies regarding associations between exposure to these animals and DNA damage. However, poultry (Beane Freeman et al. 2012) and pig (Hofmann et al. 2018) farming were associated with a decreased risk of lung cancer in the AHS cohort, whereas such associations were not present in the AGRICAN cohort (Tual et al. 2017). Additionally, raising poultry was associated with a greater risk of colorectal cancer (CRC) in AHS (Beane Freeman et al. 2012) and AGRICAN (Talibov et al. 2022), and pig farming with an increase in meningioma (Piel et al. 2017) and CRC risk (Talibov et al. 2022) in AGRICAN, although this last association does not last when only women are considered.

A larger surface of meadows on the farm was associated with less DNA damage, our study is the first to report such association. A study reported an increased risk of glioma for workers performing tasks associated with meadows (Piel et al. 2017), but the association we report is associated the presence of meadows, not performing tasks on this crop.

At this stage, it would be arbitrary to infer any causal relationship between the alterations in DNA damage we observe and a specific cancer risk. Moreover, our study, unlike most others, is reporting results specific for women, who can experience cancer risks different from men for similarly reported exposures, such as for CRC and pig farming (Talibov et al. 2022), or for bladder cancer where the risk reported in women is higher than in men for field-grown vegetables workers (Boulanger et al. 2017). If anything, observed modifications in the level of DNA damage associated with exposure to meadows, poultry and pig farming, underline that these exposures do impact women biology at the level of DNA damage in circulating PBMC, and hence alter genome stability. This warrant further studies on female cohorts to better characterize their exposure, by questionnaire and/or biomarkers of exposure. The study of additional markers linked with genome stability and DNA damage, such as micronuclei or levels of DNA methylation, could help better characterize the biological consequences of these exposures. Eventually, the ability to assess cancer risk in women farmers specifically would also be desirable.

### Influence of smoking and other lifestyle factors on DNA damage

Smokers were found to have less DNA damage than non-smokers in our analysis. While smoking has been associated with increased DNA damage in some comet-based studies (Hoffmann et al. 2005), some others did not, including in a study analyzing data from more than 19,000 individuals (Milić et al. 2021). It was however underlined that most studies did not report precisely enough the smoking status or intensity of participants. Interestingly, another study reported a decrease in DNA damage, measured by micronuclei, for smokers and former smokers (Bonassi et al. 2003).

A previous study did mention an increase in DNA damage in male smoking farmers (Lebailly et al. 1998b). It is thus possible that either different tasks performed on farms and/or sex are influencing the DNA damage to PBMC induced by smoking.

Some studies suggested that females repair DNA damage less efficiently than males (Wei et al. 2000), while others did find no difference (Soares et al. 2014); thus preventing us to attribute an effect of sex on our association.

However, our observation of a lesser extent of DNA damage decrease in former smokers compared to smokers echoes with a recent study exploring the changes in DNA methylation induced by smoking in blood samples from 745 women, where smoking-associated changes were slowly returning to normal after smoking cessation over years (Guida et al. 2015). Because PBMCs are short-lived, former smoking cannot affect them directly. It is however possible that smoking may have affected the repair capacities of progenitor cells for PBMC at the epigenetic level, which would therefore be passed on to PBMC daughter cells, even after smoking cessation.

Further studies exploring DNA repair capacities and epigenetic status in women with regard to smoking would be warranted to explore these aspects.

## Concluding remarks

To our knowledge, no other study reported this repertoire of specific tasks and exposures associated with DNA damage in a cohort of over 200 female agricultural workers, whom are only rarely studied whereas they experience specific exposures. We report an absence of effect of exposure to several potentially harmful chemicals on DNA damage, as well as alteration in the level of DNA damage for active and former smokers, and for women environmentally exposed to meadows, pig, and poultry farming. These results underline the need for further studies on women specific exposures in order to better assess the risks they are exposed to.

### Supplementary Information

Below is the link to the electronic supplementary material.

Supplementary file1 (DOCX 23 KB)

## Data Availability

Data will be made available upon reasonable request.
