# Supplementary material for: Agricultural exposures and DNA damage in PBMC of female farmers measured using the alkaline comet assay
Source: Int Arch Occup Environ Health. 2024 Mar 2;97(4):353–63. doi: 10.1007/s00420-024-02049-z (PMC10999382; doi:10.1007/s00420-024-02049-z)
Supplement: Supplementary file 1 — Supplementary file1 (DOCX 23 KB) [file 420_2024_2049_MOESM1_ESM.docx]

**Journal: International archives of occupational and environmental health**

**Agricultural exposures and DNA damage in PBMC of female farmers measured using the alkaline comet assay.**

Evenden P ^1,2^, Vandoolaeghe Q ^1,2^, Lecluse Y ^1,2^, Gac AC ^1,2^, Delépée R ^1,2^, Weiswald LB ^1,2^, Boutet-Robinet E ^3^, Boulanger M ^1,2^, Bonassi S ^4^, Lebailly P ^1,2^, Meryet-Figuière M* ^1,2^.

1. Normandie Univ, Université de Caen Normandie, Inserm U1086 ANTICIPE (Interdisciplinary Research Unit for Cancer Prevention and Treatment), Caen, France

2. Comprehensive Cancer Center François Baclesse, UNICANCER, Caen, France

3. Toxalim (Research Centre in Food Toxicology), INRAE, ENVT, INP-Purpan, UPS, Université de Toulouse, Toulouse, France

4. Department of Human Sciences and Quality of Life Promotion, San Raffaele University, Rome, Italy, Unit of Clinical and Molecular Epidemiology, IRCCS San Raffaele Roma, Rome, Italy.

* Corresponding author: [m.meryet-figuiere@baclesse.unicancer.fr](mailto:m.meryet-figuiere@baclesse.unicancer.fr)

***Supplementary data***

Table S1: Distribution of raw DNA damage scores without HDCs, normalized scores on the standardization control, and log transformed scores.

| Control score (n=41) | **Min** | **Q1** | **Median** | **Q3** | **Max** | **Mean** | **Std** |
| --- | --- | --- | --- | --- | --- | --- | --- |
|  | 2 | 5.14 | 8.04 | 11.57 | 21.64 | 8.77 | 4.95 |
| Score at enrolment (n=245) | 1 | 7.45 | 13.18 | 21.39 | 278.13 | 20.23 | 30.63 |
| Normalized | 0.08 | 0.83 | 1.72 | 3.16 | 57.32 | 3.47 | 6.29 |
| Log transformed | -2.51 | -0.19 | 0.54 | 1.15 | 4.05 | 0.56 | 1.10 |

Table S2: Full univariable analysis of HDC included comet scores and socio-demographic data.

| **Characteristics** | **Median comet score (IQR)** | **β coefficient ± std** | **p-value** |
| --- | --- | --- | --- |
| Age, n=245 | 49 (33) | 0.003 ± 0.004 | 0.51 |
| BMI |  |  |  |
| <18.5 (underweight), n=5 | 41 (25) | -0.53 ± 0.33 | 0.11^a^ |
| 18.5-25[ (normal), n=142 | 50.5 (33) | Ref |  |
| ≥25 (overweight) | 48 (33) | 0.04 ± 0.09 | 0.69 |
| Smoking status |  |  |  |
| Smoker, n=23 | 48 (27) | -0.26 ± 0.16 | 0.10^a^ |
| Former smoker, n=27 | 44 (30) | 0.03 ± 0.15 | 0.86 |
| Never smoker, n=195 | 50 (34) | Ref |  |
| Pack-years, n=17 | 48 (26) | -0.007 ± 0.03 | 0.84 |
| Cigarettes/day, n=45 | 48 (28) | -0.0123 ± 0.012 | 0.31 |
| Alcohol consumption |  |  |  |
| None, n=51 | 52 (19) | Ref |  |
| Occasional, n=110 | 45.5 (32) | -0.09 ± 0.12 | 0.48 |
| Daily n=82 | 50 (38) | -0.06 ± 0.13 | 0.64 |

Table S3: Full univariable analysis of HDC included comet scores and occupational tasks.

| **Exposure variable** | **Median comet score (IQR)** | **β coefficient ± std** | **p-value** |
| --- | --- | --- | --- |
| **Crops** |  |  |  |
| Ploughing work, n=9 | 54 (29) | -0.21 ± 0.24 | 0.38 |
| Sewing work, n=7 | 41 (25) | -0.08 ± 0.27 | 0.78 |
| Harvest work, n=34 | 41 (26) | -0.13 ± 0.13 | 0.34 |
| Seed treatment, n=5 | 34 (21) | -0.10 ± 0.32 | 0.74 |
| Storage silo treatment, n=6 | 36 (27) | -0.16 ± 0.29 | 0.60 |
| Use of herbicides on meadows, n=5 | 38 (18) | 0.10 ± 0.33 | 0.76 |
| Duration of use, n=5 |  | 0.05 ± 0.02 | 0.10^a^ |
| **Livestock** |  |  |  |
| Care of livestock, n=159 | 48 (34) | -0.12 ± 0.10 | 0.23 |
| Use of hormones, n=19 | 51 (37) | 0.03 ± 0.17 | 0.87 |
| Use of antiparasites and insecticides on livestock, n=138 | 50 (33) | -0.11 ± 0.09 | 0.25 |
| Duration of use, n=126 | 48 (33) | -0.00002 ± 0.01 | 0.99 |
| Milking, n=109 | 52 (34) | 0.13 ± 0.09 | 0.18^a^ |
| Disinfection of milking equipment, n=117 | 52 (34) | 0.03 ± 0.09 | 0.78 |
| Duration of use, n=112 | 52 (35) | 0.004 ± 0.008 | 0.56 |
| **Other** |  |  |  |
| Driving of other machinery, n=7 | 43 (26) | -0.32 ± 0.27 | 0.23 |
| Rodent poison (raticide, etc.), n=92 | 50 (35) | -0.08 ± 0.10 | 0.43 |
| Use of herbicides on courtyards, n=113 | 48.5 (35) | -0.07 ± 0.09 | 0.42 |
| Duration of use, median (IQR), n=103 | 49 (36) | 0.01 ± 0.01 | 0.24 |
| Use of herbicides on embankments, n=32 | 48 (33) | -0.08 ± 0.14 | 0.56 |
| Duration of use, median (IQR), n=32 |  | -0.001 ± 0.01 | 0.92 |
| Disinfection of premises and facilities, n=94 | 51 (32) | 0.06 ± 0.09 | 0.52 |
| Duration of use, median (IQR), n=80 | 52 (33) | 0.01 ± 0.01 | 0.28 |
| Cleaning and upkeep of agricultural equipment, n=18 | 52.5 (39) | -0.06 ± 0.18 | 0.73 |
| Duration of use, median (IQR), n=18 |  | 0.05 ± 0.02 | 0.02* |

Table S4: Full analysis of farm characteristics and univariable association with HDC included DNA damage score.

| **Variable** |  | **Median comet score (IQR)** | **β coefficient ± std** | **p-value** | **R²** |
| --- | --- | --- | --- | --- | --- |
| Utilized agricultural area (ha), median (IQR) | 68 (65) | 0.45 (0.72) | -0.001 ± 0.001 | 0.26 | 0.01 |
| Wheat |  |  |  |  |  |
| Presence n(%) | 172 (70.20) | 0.44 (0.75) | -0.03 ± 0.10 | 0.74 | 0.001 |
| Area (ha), median (IQR) | 15.5 (23.25) | 0.44 (0.75) | -0.002 ± 0.002 | 0.39 | 0.004 |
| Barley |  |  |  |  |  |
| Presence n(%) | 102 (41.63) | 0.44 (0.77) | -0.01 ± 0.09 | 0.89 | 0.0001 |
| Area (ha), median (IQR) | 5.25 (7.50) | 0.44 (0.77) | 0.005 ± 0.01 | 0.63 | 0.002 |
| Corn |  |  |  |  |  |
| Presence n(%) | 153 (62.45) | 0.48 (0.75) | 0.08 ± 0.09 | 0.40 | 0.003 |
| Area (ha), median (IQR) | 15 (10.5) | 0.48 (0.75) | 0.003 ± 0.01 | 0.65 | 0.001 |
| Peas |  |  |  |  |  |
| Presence n(%) | 69 (28.16) | 0.40 (0.85) | -0.07 ± 0.10 | 0.50 | 0.002 |
| Area (ha), median (IQR) | 12 (17) | 0.40 (0.85) | -0.004 ± 0.007 | 0.58 | 0.005 |
| Beets |  |  |  |  |  |
| Presence n(%) | 50 (20.41) | 0.41 (0.91) | -0.05 ± 0.11 | 0.64 | 0.001 |
| Area (ha), median (IQR) | 4.25 (6) | 0.41 (0.91) | 0.001 ± 0.02 | 0.97 | 0.00002 |
| Flax |  |  |  |  |  |
| Presence n(%) | 21 (8.57) | 0.24 (0.99) | -0.23 ± 0.16 | 0.16^a^ | 0.01 |
| Area (ha), median (IQR) | 6 (5) | 0.24 (0.99) | 0.03 ± 0.02 | 0.11^a^ | 0.13 |
| Rape |  |  |  |  |  |
| Presence n(%) | 37 (15.10) | 0.42 (0.93) | -0.04 ± 0.13 | 0.77 | 0.0003 |
| Area (ha), median (IQR) | 5 (7) | 0.42 (0.93) | -0.05 ± 0.02 | 0.04* | 0.11 |
| Potato |  |  |  |  |  |
| Presence n(%) | 9 (3.67) | 0.36 (0.56) | -0.10 ± 0.24 | 0.69 | 0.001 |
| Area (ha), median (IQR) | 5 (13) | 0.36 (0.56) | 0.001 ± 0.03 | 0.97 | 0.0003 |
| Orchard |  |  |  |  |  |
| Presence n(%) | 13 (5.31) | 0.42 (0.58) | -0.03 ± 0.21 | 0.89 | 0.0001 |
| Area (ha), median (IQR) | 10 (14) | 0.42 (0.58) | 0.01 ± 0.01 | 0.19^a^ | 0.15 |
| Meadows |  |  |  |  |  |
| Presence n(%) | 220 (89.80) | 0.44 (0.71) | -0.12 ± 0.15 | 0.43 | 0.003 |
| Area (ha), median (IQR) | 25 (27) | 0.44 (0.71) | -0.003 ± 0.002 | 0.14^a^ | 0.01 |
| Livestock farming |  |  |  |  |  |
| Presence n(%) | 239 (97.55) | 0.45 (0.71) | -0.19 ± 0.30 | 0.52 | 0.002 |
| Dairy |  |  |  |  |  |
| Presence n(%) | 149 (60.82) | 0.47 (0.75) | 0.07 ± 0.10 | 0.49 | 0.002 |
| Cattle |  |  |  |  |  |
| Presence n(%) | 207 (84.49) | 0.45 (0.71) | 0.02 ± 0.13 | 0.87 | 0.0001 |
| Number of animals, median (IQR) | 100 (85) | 0.45 (0.71) | 0.0004 ± 0.001 | 0.15^a^ | 0.01 |
| Sheep |  |  |  |  |  |
| Presence n(%) | 57 (23.27) | 0.39 (0.68) | -0.08 ± 0.11 | 0.44 | 0.002 |
| Number of animals, median (IQR) | 5 (7) | 0.39 (0.68) | -0.004 ± 0.002 | 0.02* | 0.09 |
| Pig |  |  |  |  |  |
| Presence n(%) | 41 (16.73) | 0.61 (0.68) | 0.19 ± 0.12 | 0.12^a^ | 0.01 |
| Number of animals, median (IQR) | 2 (2) | 0.61 (0.68) | -0.0003 ± 0.0003 | 0.24 | 0.02 |
| Horse |  |  |  |  |  |
| Presence n(%) | 49 (20.00) | 0.39 (0.62) | -0.08 ± 0.11 | 0.49 | 0.004 |
| Number of animals, median (IQR) | 3 (9) | 0.39 (0.62) | 0.002 ± 0.01 | 0.80 | 0.001 |
| Goat |  |  |  |  |  |
| Presence n(%) | 17 (6.94) | 0.57 (0.54) | 0.13 ± 0.18 | 0.47 | 0.002 |
| Number of animals, median (IQR) | 1 (1) | 0.57 (0.54) | -0.08 ± 0.11 | 0.48 | 0.03 |
| Rabbit |  |  |  |  |  |
| Presence n(%) | 98 (40.00) | 0.47 (0.64) | 0.03 ± 0.09 | 0.74 | 0.001 |
| Number of animals, median (IQR) | 10 (11) | 0.47 (0.64) | -0.0003 ± 0.0002 | 0.13^a^ | 0.02 |
| Poultry |  |  |  |  |  |
| Presence n(%) | 183 (74.69) | 0.42 (0.67) | -0.10 ± 0.11 | 0.34 | 0.004 |
| Number of animals, median (IQR) | 30 (35) | 0.42 (0.67) | 0.00001 ± 0.00001 | 0.28 | 0.01 |
